# FRAX-Based Intervention Thresholds for Osteoporosis Treatment in Ukraine

**DOI:** 10.1155/2021/2043479

**Published:** 2021-06-10

**Authors:** Vladyslav Povoroznyuk, Nataliia Grygorieva, Helena Johansson, Mattias Lorentzon, Nicholas C Harvey, Eugene V McCloskey, Anna Musienko, Enwu Liu, John A Kanis, Nataliia Zaverukha, Oksana Ivanyk

**Affiliations:** ^1^State Institution, D.F. Chebotarev Institute of Gerontology NAMS of Ukraine, Kyiv, Ukraine; ^2^Centre for Metabolic Bone Diseases, University of Sheffield, Sheffield, UK; ^3^Mary McKillop Health Institute, Australian Catholic University, Melbourne, Australia; ^4^Geriatric Medicine, Department of Internal Medicine and Clinical Nutrition, Institute of Medicine and Clinical Nutrition, Sahlgrenska Academy, University of Gothenburg, Gothenburg, Sweden; ^5^MRC Lifecourse Epidemiology Unit, University of Southampton, Southampton, UK; ^6^Centre for Metabolic Bone Diseases, University of Sheffield Medical School, Sheffield, UK; ^7^MRC and Arthritis Research UK Centre for Integrated Research in Musculoskeletal Ageing, Mellanby Centre for Bone Research, University of Sheffield, Sheffield, UK

## Abstract

**Objectives:**

Osteoporosis, in addition to its consequent fracture burden, is a common and costly condition. FRAX^®^ is a well-established, validated, web-based tool which calculates the 10-year probability of fragility fractures. A FRAX model for Ukraine has been available since 2016 but its output has not yet been translated into intervention thresholds for the treatment of osteoporosis in Ukraine; we aimed to address this unmet need in this analysis.

**Methods:**

In a referral population sample of 3790 Ukrainian women, 10-year probabilities of major osteoporotic fracture (MOF) and hip fracture separately were calculated using the Ukrainian FRAX model, with and without femoral neck bone mineral density (BMD). We used a similar approach to that first proposed by the UK National Osteoporosis Guideline Group, whereby treatment is indicated if the probability equals or exceeds that of a woman of the same age with a prior fracture.

**Results:**

The MOF intervention threshold in females (the age-specific 10-year fracture probability) increased with age from 5.5% at the age of 40 years to 11% at the age of 75 years where it plateaued and then decreased slightly at age 90 (10%). Lower and upper thresholds were also defined to determine the need for BMD, if not already measured; the approach targets BMD measurements to those at or near the intervention threshold. The proportion of the referral populations eligible for treatment, based on prior fracture or similar or greater probability, ranged from 44% to 69% depending on age. The prevalence of the previous fracture rose with age, as did the proportion eligible for treatment. In contrast, the requirement for BMD testing decreased with age.

**Conclusions:**

The present study describes the development and application of FRAX-based assessment guidelines in Ukraine. The thresholds can be used in the presence or absence of access to BMD and optimize the use of BMD where access is restricted.

## 1. Introduction

The burden of osteoporosis and its related fractures is increasingly recognised; for example, the number of years lived with a disability is greater for osteoporosis than for any single cancer, except for lung cancer, and is comparable to or greater than that due to many chronic noncommunicable diseases [1, 2]. An estimated 2.7 million hip fractures occurred in 2010 worldwide [3], with around 620,000 hip and 1.8 million other fragility fractures in the EU in the same year [4, 5]. The total cost in the EU, including values of quality-adjusted life years (QALYs) lost, was estimated at €98 billion, a figure that is expected to rise to €121 billion in 2025. Among recent developments in the management of osteoporosis, the use of tools designed to calculate fracture risk is increasingly adopted to improve the identification of those at the highest risk who would benefit from appropriate treatment. Of these, the widely used FRAX^®^ tool (http://www.sheffield.ac.uk/FRAX) computes the 10-year probability of a major osteoporotic fracture (MOF, comprising a hip, spine, forearm, or humerus fractures) or hip fracture alone from simple, easily captured clinical risk factors (CRFs) with the optional incorporation of femoral neck bone mineral density (BMD) measured by dual-energy Х-ray absorptiometry (DXA) [6, 7]. It is increasingly incorporated within national and international guidelines for osteoporosis [8] with some countries providing direct linkages between the FRAX assessment and clinical guidance websites, for example, in the UK [9, 10]. A FRAX calculator, calibrated to the epidemiology of hip fracture and mortality in Ukraine, was launched in 2016 but has not yet been linked to intervention thresholds [11].

A number of approaches have been taken in the development of intervention thresholds and are discussed elsewhere [8]. In brief, within most guidelines, it is recommended that treatment can be considered in postmenopausal women with a history of fracture, regardless of BMD, with some guidelines restricting this to previous vertebral or hip fracture. If such patients are eligible for treatment, then those at similar or greater risk, but without a previous fracture, should also be considered for treatment. This approach, first promoted by the National Osteoporosis Guideline Group in the UK and subsequently adopted in European guidance, produces an age-dependent threshold as the 10-year probability of fracture is in itself age-dependent [10, 12–14]. Given the well-described differences in fracture epidemiology across countries, the thresholds should be calibrated to the fracture risk within each country. In this analysis, we wished to develop these thresholds for Ukraine and explore their use and impact within a Ukrainian referral population.

## 2. Methods

### 2.1. Development of Intervention and Assessment Thresholds

The 10-year probabilities of MOF and hip fracture were calculated using the Ukrainian model of FRAX (version 4.1). Given the assumption that a prior fracture was considered to carry a sufficient risk to recommend treatment, the intervention threshold for women without a prior fracture was set at the age-specific 10-year probability of a MOF equivalent to women with a previous fragility fracture, but without any other clinical risk factors. At all ages, the body mass index (BMI) was set to 25 kg/m^2^.

In addition to the intervention threshold, two assessment thresholds were derived to enable the clinician to consider the need for BMD assessment, if the latter had not already been performed [10]. These assessment thresholds, again based on MOF probabilities, comprised the following.

A lower threshold of probability: this was defined as the probability of fracture in a woman with BMI of 25 kg/m^2^ but without any clinical risk factors, that is, in the absence of such risk factors, there is no need to consider a BMD test or treatment.

An upper threshold of probability: defined as a probability above which treatment could be recommended without necessarily measuring BMD. To minimise the likelihood that a woman at high risk of fracture, based on clinical risk factors alone, would be reclassified to the group with low risk following a BMD test, the threshold was set at 1.2 times the intervention threshold.

Application of this guideline then results in 4 groups of patients at first assessment (i.e., before any input from BMD measurements). The first group comprises women with a previous fragility fracture to be considered as eligible for treatment without any need for further assessment (BMD could be used to monitor treatment response). The second group consists of those women who, following the FRAX assessment, have low probabilities that lie below the lower assessment threshold so that no further action is required. The third group comprises women without a history of fracture but with high fracture probability above the upper assessment threshold in whom treatment should also be considered. The final group are those women who, in the absence of the previous fracture, have a FRAX MOF probability falling between the lower and upper assessment thresholds in whom a BMD measurement is mandated with the fracture probability then recalculated with the inclusion of this additional information. On recalculation, individuals are classified as eligible for treatment when the probability lies above the intervention threshold.

### 2.2. Impact of Thresholds

The application of these thresholds was then tested in a referral population of 3790 women attending the D.F. Chebotarev Institute of Gerontology in Ukraine for the assessment of osteoporosis between May 2017 and May 2019. The study was approved by the Ethics Committee of the D.F. Chebotarev Institute of Gerontology NAMS of Ukraine (17/05/2017, protocol no. 5) and all participants provided written informed consent. Bone density was measured on Hologic or GE Lunar equipment, with the T-scores converted to Hologic T-score units using established conversion equations.

### 2.3. Statistical Analysis

The analysis was performed by Statistica 10.0 software. The sample's relevance in terms of the normal distribution principle was checked by Shapiro–Wilk's test. The data were presented as *n (%)* and also as mean values *(M)* and standard deviation *(SD)* or median *(Ме)* and the lower and upper quartiles *(25Q*–*75Q)* according to data distribution.

## 3. Results

**Figure 1:**
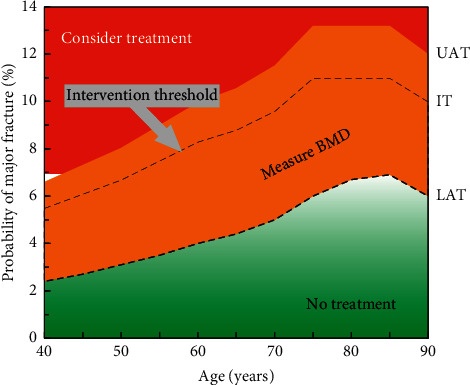
Intervention (IT), lower (LAT), and upper (UAT) assessment thresholds based on major osteoporotic fracture probabilities for use with the FRAX tool for Ukraine. *Note*. Values within the red area merit consideration of treatment without the need for BMD, while those in the green area suggest that no further assessment (e.g., BMD) is necessary. Values in the orange area, around the IT, indicate that femoral neck BMD assessment and incorporation of the result into the FRAX calculation is warranted.

The intervention and assessment thresholds, derived from FRAX MOF probabilities using the Ukraine calculator, are shown in Table 1 and Figure 1. The intervention threshold increased with age from 5.5% at the age of 40 years to 11% at the age of 75 years, with a subsequent plateau at age 85 years and a small decrease thereafter. The upper and lower assessment thresholds also varied with age and provided guidance on the need for the measurement of BMD in the assessment of fracture probability. For example, at the age of 60 years and in the absence of a prior fracture, a BMD test would be recommended in an individual with a fracture probability that lay between 4% and 10%. Following recalculation of the FRAX MOF probability with the inclusion of the femoral neck BMD T-score, treatment would be recommended if the MOF probability was 8.3% or greater.

### 3.1. Impact of Thresholds

The baseline characteristics of the referral population, comprising 3790 women (85% postmenopausal), are shown in Table 2. The mean age was 62 years and the mean BMI was 27.8 kg/m^2^. Prior fracture was by far the most common clinical risk factor (51.5%), whereas smoking was very uncommon.

**Figure 2:**
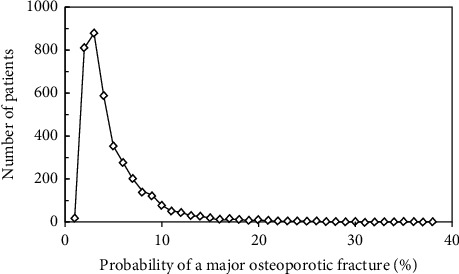
The distribution of FRAX MOF probabilities in the referral cohort of 3719 women. In this example, FRAX MOF probability is calculated with the inclusion of femoral neck BMD.

FRAX probabilities could be calculated in 3719 women (98.1%) due to a small number of women not having clear information about the causes of secondary osteoporosis. The mean probability of a MOF was 6.0%, and for a hip fracture, it was 1.3% when BMD was not included in the FRAX calculation. FRAX probabilities for both outputs were higher when an incident of fracture was included in the calculation (Table 3). All probabilities showed a skewed distribution as illustrated for the output of MOF FRAX probability calculated with BMD (Figure 2).

**Figure 3:**
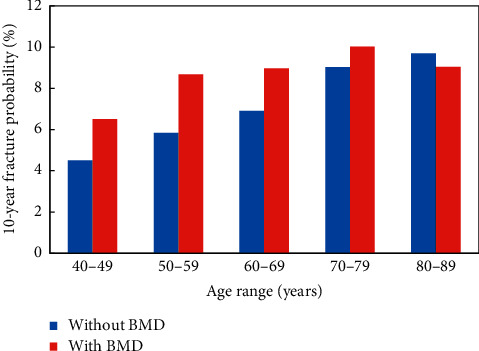
Mean FRAX MOF probabilities, calculated with and without femoral neck BMD, across the age categories within the referral population.

Within the referral cohort, mean FRAX MOF probabilities largely increased progressively with age (Figure 3). For example, the mean probability calculated without BMD was 4.4% at age of 40–49 years and was twofold higher at the age of 80–89 years. Mean FRAX MOF probabilities calculated with BMD were higher than those calculated without BMD in most age groups; this was particularly noted at younger ages, decreased at older ages, and was reversed in those aged 80–89 years.

**Figure 4:**
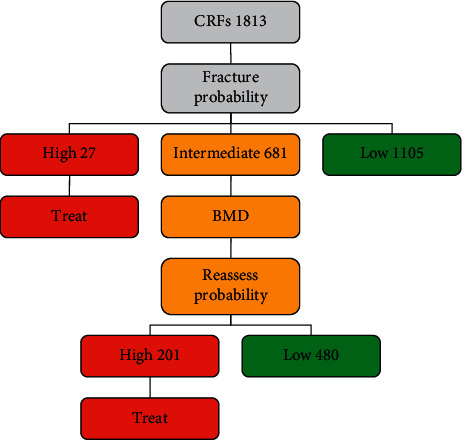
Application of the Ukrainian FRAX MOF assessment and intervention thresholds to the referral population. The numbers in each category of risk denote the number of women in each category.

When applying the thresholds as described in the methods to derive the categories of risk, the 1906 women with prior fracture (51.3% of the 3719 women assessed) were classified as needing treatment on this basis. Of the women without prior fracture (*n* = 1813), only a small number (27 women, 0.7% of the whole cohort assessed) had sufficiently high FRAX MOF probabilities to exceed the upper assessment threshold (Figure 4). In contrast, a significant proportion (1105 women, 29.7% of the cohort) were found to have FRAX MOF probabilities that lay below the lower assessment threshold, suggesting that no further assessment (including BMD) would be required. Thus, the intermediate category of risk comprised 681 women (18.3% of the whole cohort assessed) in whom BMD would be assessed and FRAX recalculated with the inclusion of femoral neck BMD (Figure 4). Of these, 480 women (12.9% of the whole cohort assessed) had final FRAX MOF probabilities that fell below the intervention threshold (treatment not recommended), whereas 201 women (5.4% of the cohort) had values at or above the intervention threshold and should be recommended treatment. At the end of the process, a total of 2134 women (57.4%) would be recommended treatment (Figure 4).

**Figure 5:**
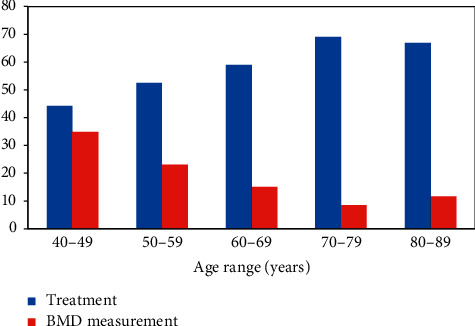
Proportion of women recommended treatment and the proportion needing BMD tests, by age, when the guidance and Ukrainian FRAX thresholds are applied to the referral population, %.

The final disposition of the cohort in terms of treatment being recommended and the need for BMD testing is shown in Figure 5. The proportion requiring treatment increased from 44% in women of ages 40–49 years to 69% in those of ages 70–79 years. Within each age group, a prior fracture was by far the major factor determining the need for treatment. The use of the guidance and thresholds resulted in a low overall need for BMD testing (19% of the whole cohort as reported above), but this was also age-dependent with a greater proportion of the younger age groups needing BMD compared to a much lower proportion of the older age groups (Figure 5).

## 4. Discussion

In this report, we present potential guidelines and thresholds for the use of 10-year fracture probability by FRAX in clinical practice in Ukraine. The thresholds are driven by a combination of the epidemiology of fracture in Ukraine, reflected within the FRAX calculator for Ukraine, and a method for determining intervention thresholds proposed within European guidance [11, 14]. The approach provides for a more restricted but targeted use of DXA-measured BMD which is particularly useful if access to BMD is limited; the thresholds can indeed be used in the complete absence of BMD so that patients in regions with limited resources are not disenfranchised. The simple rationale is that if postmenopausal women, of any age, with a previous fragility fracture, can be recommended treatment to reduce future fracture risk, as is commonly considered, then an individual without fracture but with equivalent fracture probability should also be eligible for treatment.

The combination of risk assessment and treatment consideration leads to an easily applicable clinical pathway. The starting point is the recognition of the presence of one or more risk factors in an individual which then triggers the physician to consider osteoporosis and, more importantly, fracture risk. This case-finding strategy has long been promoted in the majority of osteoporosis guidelines, but in the past led to the advice that BMD measurements should subsequently be obtained in all patients, with treatment indicated on the finding of BMD-defined osteoporosis (lumbar spine or hip BMD T-score < -2.5) [15]. The role of detecting BMD-defined osteoporosis was twofold. Firstly, the evidence base for osteoporosis treatments had largely been built on randomized clinical trials with BMD-defined osteoporosis as an entry criterion; this led to the belief by many that treatments would not work in the absence of BMD-defined osteoporosis. This is now rightly regarded as an incorrect interpretation of the data with several recent studies confirming that osteoporosis treatments can reduce fracture risk in patients with BMD above the osteoporosis threshold [16]. For example, in a 6-year study where women with osteopenia were randomized to receive 18-monthly infusions of zoledronate or placebo, osteoporotic fracture risk was reduced by 37% [17]. In addition, a population-based UK study of screening for high hip fracture risk using FRAX showed an overall 28% reduction in hip fractures, despite half of the treated group having hip BMD above the osteoporosis range [18]. The second reason for the detection of BMD-defined osteoporosis was the frequent requirement in many countries of a BMD-defined “diagnosis” of osteoporosis for reimbursement of any treatment costs. The move of clinicians away from the dependence on BMD to the management of patients based on fracture risk is an issue that requires further discussion between osteoporosis specialists, healthcare providers, and payers in the coming months and years.

In the new clinical pathway, the presence of the clinical risk factor now mandates a fracture risk assessment by FRAX, with the measurement of BMD viewed as an additional risk factor to be assessed in all patients or in a subset. For countries with less than optimal DXA provision, such as the UK and many other European countries, the optimal use of DXA is to limit its use to patients in whom the result might impact on the decision to treat or not, that is, those with FRAX probabilities lying at or near an intervention threshold. In the current study, the proportion of the referral population requiring scans was substantially reduced from 48.3%, representing all of those without fracture, to only 19% when confined to those with FRAX probabilities lying between the upper and lower assessment thresholds. At a population level in the UK, it has been shown that this more targeted use of BMD identifies only a slightly reduced proportion of women at high risk (average 34.6% vs. 35.7% across all ages), but with lower numbers of scans required at each age [19]. For example, it required only 3.5 scans at the age of 50 years to identify one case of hip fracture, whereas the BMD measurement in all required 13.9 scans per identified hip fracture. The acquisition costs for identifying a hip fracture case and the total costs (acquisition and treatment) per hip fracture averted were lower [19].

Our analysis has one main limitation. The FRAX outputs are calibrated to the best available data on fracture and mortality within the country of interest; assessment of its performance is then best performed when the sample reflects the overall population, whereas the current study is based on a referral population that is highly unlikely to be representative. This is reflected in the very high prevalence of fracture in the studied cohort and, as expected, this is then the major driver of the decision to treat (51.3%), with the FRAX assessment only adding a modest number of additional women needing treatment (6.1% of whole cohort or 11.9% of those treated). It is likely that the latter would be of higher relative importance if, for example, a true population screening program was undertaken. In the SCOOP study of population screening in the UK, where only 22% of women had a history of prior fracture, the proportion identified for treatment in the absence of fracture was 7.8% of the whole cohort or 26% of those recommended for treatment [18]. This rises still further when, as in the SCOOP study, the prior fracture is handled as a risk factor rather than an independent criterion for treatment; in the final high-risk category of the SCOOP study, 54.5% of the women were categorized as such in the absence of a prior fracture [18]. These considerations suggest that the low prevalence of women eligible for treatment is a function of the atypical referral population rather than in the epidemiology of fracture in Ukraine. On a separate note, while this use of FRAX-based intervention in the setting of a case-finding approach has been shown to be cost-effective in a UK setting [20], cost-effectiveness is likely to differ in Ukraine because of different fracture risks and costs. Further studies and analyses in nonreferral populations in Ukraine would be useful to further characterize the performance of the intervention and assessment thresholds.

## 5. Conclusion

The present study describes the establishment of intervention and assessment thresholds for FRAX-based fracture risk assessment in Ukraine. Implementation of the approach described here would enable appropriate treatment of women at high risk of fracture with a potential substantial reduction in the use of BMD resources for risk assessment (the latter may still be needed for monitoring of treatment response depending on local guidance). The study highlights potential disparities between intervention thresholds and reimbursement criteria and this will need resolution, possibly by underpinning these guidelines with an economic assessment. Overcoming these hurdles is, however, very likely to improve the delivery of health care to those most in need with high fracture risk.

## Figures and Tables

**Table 1:** Values of 10-year FRAX MOF probability (%), calculated without BMD using the Ukrainian model of FRAX, for the lower, upper, and intervention thresholds by age.

Age (years)	Lower assessment threshold	Upper assessment threshold	Intervention threshold^#^
40	2.4	6.6	5.5
45	2.7	7.3	6.1
50	3.1	8.1	6.7
55	3.5	9.1	7.5
60	4.0	10	8.3
65	4.4	11	8.8
70	5.0	12	9.6
75	6.0	13	11
80	6.7	13	11
85	6.9	13	11
90	6.0	12	10

*Note*. ^#^The Intervention threshold is the probability of a MOF for a female with a BMI of 25.0 kg/m^2^ and a prior fragility fracture without other clinical risk factors and BMD.

**Table 2:** Characteristics of the referral population.

Variable (description)	Mean ± SD (range) or *n* (%)
Age (years)	62.0 ± 10.0 (40–90)
Height (cm)	162.1 ± 6.5 (130–187)
Weight (kg)	73.1 ± 14.5 (39–140)
Body mass index (kg/m^2^)	27.8 ± 5.3 (13.9–54.1)
Postmenopausal status, *n* (%)	3209 (84.7%)
Previous fracture, *n* (%)	1950 (51.5%)
Parental fracture hip, *n* (%)	165 (4.4%)
Current smoking, *n* (%)	4 (0.0%)
Glucocorticoids, *n* (%)	293 (7.7%)
Rheumatoid arthritis, *n* (%)	211 (5.6%)
Secondary osteoporosis, *n* (%)	83 (2.4%)
Alcohol (3 or more units per day), *n* (%)	63 (1.7%)
BMD of femoral neck (T-score at baseline^*a*,*b*^)	−1.7 ± 1.5 (−5.4–4.3)

^*a*^For Hologic: T-score = (BMD–0.858)/0.120 [9]. ^*b*^For GE Lunar: T-score = ((−0.023 + 0.939 × BMD–0.019)/1.087–0.858)/0.120) [9, 11].

**Table 3:** Ten-year probability of the fractures in the studied population.

FRAX Ten-year probability	All group	Women without fractures	Women with fractures
MOF calculated without BMD (%)	6.0 [3.6–8.7]	3.7 [3.1–4.7]	8.4 [6.9–10.0]
Hip fracture calculated without BMD (%)	1.3 [0.5–2.4]	0.6 [0.3–1.1]	2.1 [1.3–3.4]
MOF calculated with BMD (%)	5.9 [3.8–9.2]	3.9 [3.1–5.3]	8.6 [6.3–12.0]
Hip fracture calculated with BMD (%)	1.1 [0.4–2.6]	0.6 [0.2–1.3]	2.1 [1.0–3.9]

Data are presented in Me [Q25-Q75].

## Data Availability

All the data used to support the results of this study are stored at and available from the corresponding author on request.
